# Nuclear receptors: a bridge linking the gut microbiome and the host

**DOI:** 10.1186/s10020-021-00407-y

**Published:** 2021-11-05

**Authors:** Zixuan Wang, Wei-Dong Chen, Yan-Dong Wang

**Affiliations:** 1 State Key Laboratory of Chemical Resource Engineering, College of Life Science and Technology, Beijing University of Chemical Technology, Beijing, People’s Republic of China; 2 Key Laboratory of Molecular Pathology, Key Laboratory of Receptors-Mediated Gene Regulation and Drug Discovery, School of Basic Medical Science, Inner Mongolia Medical University, Hohhot, Inner Mongolia People’s Republic of China; 3 School of Medicine, Key Laboratory of Receptors-Mediated Gene Regulation, The People’ Hospital of Hebi, Henan University, Henan, People’s Republic of China

**Keywords:** Gut microbiome, Nuclear receptors, Inflammatory bowel disease, Obesity, Diabetes

## Abstract

**Background:**

The gut microbiome is the totality of microorganisms, bacteria, viruses, protozoa, and fungi within the gastrointestinal tract. The gut microbiome plays key roles in various physiological and pathological processes through regulating varieties of metabolic factors such as short-chain fatty acids, bile acids and amino acids. Nuclear receptors, as metabolic mediators, act as a series of intermediates between the microbiome and the host and help the microbiome regulate diverse processes in the host. Recently, nuclear receptors such as farnesoid X receptor, peroxisome proliferator-activated receptors, aryl hydrocarbon receptor and vitamin D receptor have been identified as key regulators of the microbiome-host crosstalk. These nuclear receptors regulate metabolic processes, immune activity, autophagy, non-alcoholic and alcoholic fatty liver disease, inflammatory bowel disease, cancer, obesity, and type-2 diabetes.

**Conclusion:**

In this review, we have summarized the functions of the nuclear receptors in the gut microbiome-host axis in different physiological and pathological conditions, indicating that the nuclear receptors may be the good targets for treatment of different diseases through the crosstalk with the gut microbiome.

## Introduction

The gut microbiome is the totality of microorganisms, bacteria, viruses, protozoa, and fungi within the gastrointestinal tract (Corrigan et al. 2018). And it plays various roles in different physiological and pathological conditions (Backhed et al. 2005). The composition of the microbiome in the intestine is diverse and depends upon the environment, gender, diet, age, immune system, xenobiotic exposure, etc. (Feng et al. 2018). In recent years, an increasing number of reports have shown the interactions between the host and microbiome but the underlying mechanisms remain unclear (Tremaroli and Backhed 2012). These interactions are involved in various processes such as metabolism (Federici 2019), immunomodulation (du Teil Espina et al. 2019) and autophagy (Jin et al. 2015). The gut microbiome affects host physiology and host condition alters the gut microbiome composition. For example, the phenotype of high-fat-diet-induced weight gain will be transferred to germ-free mice through fecal microbiota transplant, indicating the gut microbiome affects the host while some gene knockout mice have the altered gut microbiota compared with wild-type mice (Parséus et al. 2017). These reports indicate that the gut microbiome can be regarded as a subsystem in the intestine. The gut microbiome is related to various diseases such as obesity, diabetes, clinic inflammation and cancer. The gut microbiome affects nuclear receptors (NRs) through a variety of factors such as bile acids (BAs), short-fatty acids (SCFAs) and Vitamins. And the roles of NRs in various pathological and physiological processes of the host can be changed by this effect. In other words, NRs play different roles as intermediaries or a bridge between the gut microbiome and the host.

NRs are a group of ligand-binding transcription factors and mediators of various metabolic and signaling pathways (Chawla et al. 2001). NR superfamily includes various members such as farnesoid X receptor (FXR), Liver X receptor (LXR), retinoid X receptor (RXR), pregnane and xenobiotic receptor (PXR), peroxisome proliferator-activated receptors (PPARs), constitutive androstane receptor (CAR), Vitamin D receptor (VDR), and aryl hydrocarbon receptor (AHR) (Cave et al. 2016; Zenata and Vrzal 2017; Murray et al. 2014). The abnormal states of NRs may lead to serious consequences (Lazar 2017). For example, knockout or low activation of FXR will cause a significant reduction in the rate of liver regeneration, and FXR knockout mice show higher tumor incidence (Wang et al. 2008a). These characteristic functions of NRs imply that they could be potential therapeutic targets in many diseases.

The correlation between the gene expression of the host and the composition of the microbiota has been reported increasingly often (Kurilshikov et al. 2017), indicating that the gut microbiota may have some relationships with transcription factors such as NRs. For example, SCFAs are the products of the gut microbiota and can activate PPARγ in the colon and regulate the process of inflammatory bowel disease (IBD) (Viladomiu et al. 2013). Meanwhile, the microbiota compositions are associated with endogenous factors including host-produced BAs regulated by FXR (Zheng et al. 2017). These reports indicate that NRs-microbiota axis plays key roles in the whole metabolic and signaling system. To date, FXR (Shapiro et al. 2018), AHR (Hubbard et al. 2015), VDR (Wang et al. 2016a), and PPARs (Mishra et al. 2016) have been confirmed to be closely related to the gut microbiota. It suggests that NRs could be identified as bridges between the gut microbiota and the host system.

In this review, we discussed that the physiological and pathological implications of NRs (mainly FXR, AHR, VDR, and PPARs)-gut microbiota axis and these functions are not limited to the intestine but also can be found in other organs.

## NRs: the intermediary of the host and gut microbiome

NRs have been identified as the intermediaries between the gut microbiota and the host system, even making the microbiota as an essential “independent organ” (Wahlstrom et al. 2016). In other words, NRs could be identified as a family of molecular messengers for the gut microbiota to interact with the host system (Arulampalam et al. 2006). For example, numerous genera or species of the gut microbiome can produce indole (e.g. *E.coli*) or SCFAs (e.g. *Clostridium and Lactobacillus*), some stimuli such as diet can change the abundance of these genera or species to affect the levels of indole or SCFAs (Hubbard et al. 2015; Zhao et al. 2018). Indole is the ligand of AHR (Marinelli et al. 2019), and SCFAs are the ligands of AHR and PPARs (Marinelli et al. 2019; Roy et al. 2016). These NRs are associated with various host activities such as diseases including IBD, non-alcoholic fatty liver disease (NAFLD), and alcoholic liver disease (ALD) (Hendrikx et al. 2018; Jiao et al. 2018; Lamas et al. 2016a; Mir et al. 2013). The activation of PPARγ by its ligand decreases cecal lactate levels during *Salmonella enterica Typhimurium* infection (Gillis et al. 2018). And VDR can maintain the antimicrobial function of Paneth cells in the gut to maintain the gut microbiome homeostasis (Wu et al. 2015). A few of the NRs-gut microbiota crosstalk mechanisms are summarized in Table 1.

**Table 1:** NRs in the crosstalk of gut microbiome-host system

NRs	Mechanism	Diseases and phenotype	References
FXR	Tempol → *Lactobacillus*↓, BSH↓ → T-β-MCA↑ → inhibition of FXR	Obesity	Li et al. (2013)
	Gut microbiome → FXR → FGF15/19 or CYP7A1	Multi-Metabolic diseases	Al-Khaifi et al. (2018); Degirolamo et al. (2014); Gonzalez et al. (2016); Sayin et al. (2013)
	Gut microbiome → FXR → Diet-induced obesity	Obesity	Parséus et al. (2017)
	FXR↓ → ceramide↓ → SREBP-1C↓ → lipid metabolism↓, Obesity↓	Obesity, NAFLD	Gonzalez et al. (2016); Jiang et al. (2015)
	FEX → FXR → TGR5 → GLP-1 → improving glucose & insulin tolerance	T2D	Albaugh et al. (2019); Pathak et al. (2018)
	FXR↓ → butyrate producers in gut microbiome↓	NAFLD	Sheng et al. (2017)
	Gut microbiome → primary BAs change to secondary BAs → FXR-FGF pathway	IBD, NAFLD	Jiao et al. (2018)
PPARs	*Prevotella* and *Atopobium* → SCFAs → ERK1/2-PPARγ → ANGPTL4↑, ADRP↑	Epithelial damage	Nepelska et al. (2017)
	*Bacteroides* → insulin sensitive regulation	glucose metabolism	Yang et al. (2017)
	PPARα → IL-22, RegIIIβ, RegIIIγ	Gut mucosal immunity	Manoharan et al. (2016)
	Lactic acid bacteria → ALA → GPCR40 → microphage M2 differentiation	Gut mucosal immunity	Ohue-Kitano et al. (2018)
	Microbiome → lack of butyrate → absence of PPARγ signal → nitrate and lactate accumulate → exogenous infection	IBD, NAFLD	Byndloss et al. (2017); Gillis et al. (2018)
	*L.casei Zhang* → TLR-MAPK-PPARγ → inflammation↓	Liver inflammation	Wang et al. (2016b)
AHR	Ethanol → IAA-AHR-IL-22-REG3G pathway → gut bacteria transfer	Liver inflammation	Hendrikx et al. (2018)
	Trp metabolism → CARD9-AHR-IL-22	IBD	Lamas et al. (2017)
	Gut microbiome → CD4^+^-LAG3 pathway	CNS immunity	Kadowaki et al. (2016)
	AHR → RORγ + group 3 ILC	IBD	Qiu and Zhou (2013)
	ILC → inhibitor of DNA binding 2 (ID2)-AHR-IL-22 pathway or T cell	IBD	Guo et al. (2015); Wagage et al. (2015)
	Urolithin A → AHR-Nrf2 pathway	Gut barrier integrity	Singh et al. (2019)
	gut microbiome → Trp metabolism → indole derivatives → AHR-IL-22 signal → antifungal resistance and mucosal protection	Gut mucosal reactivity	Zelante et al. (2013)
	Purinergic metabolism → AHR-CD39 pathway	Immune metabolism	Longhi et al. (2017)
VDR	VD-VDR → NF-κB, MAPKs, TLR, EGFR, TJ pathways	IBD, Eystic fibrosis	Kanhere et al. (2018); Wu et al. (2010); Yoon and Sun (2011)
	VDR → Th1, Th17 cell	Mucosa inflammation, Epithelium cell apoptosis	He et al. (2018)
	*Lactobacillus casei Zhang* and Vitamin K2 → VDR → AMPK signaling pathway	Colon cancer	Zhang et al. (2017)
	VDR → ATG16L1 → autophagy	IBD, autophagy	Jin et al. (2015)

## Gut microbiota and FXR

FXR was first identified and named in 1995 (Forman et al. 1995). It belongs to a sub-cluster of receptors (including VDR, CAR, PXR, LXRα, etc.) that are metabolic regulators (Wang et al. 2008a). As a transcription factor, FXR can bind to DNA as a monomer or heterodimer with RXR and regulate the target gene expression. It, however, was identified as an orphan nuclear receptor initially (Kliewer et al. 1999; Wang et al. 2008b). The physiological ligands of FXR are BAs (Makishima et al. 1999; Wang et al. 1999), and chenodeoxycholic acid (CDCA) is a typical natural ligand of FXR (Gustafsson 1999). FXR regulates various physiological activities such as cholesterol catabolism (Russell 1999), liver regeneration (Chen et al. 2010; Zhang et al. 2012), inflammation and immunoreaction (Wang et al. 2008c), and glucose metabolism through different pathways (Pathak et al. 2018). These reports indicate that FXR is an essential regulator in vivo.

The earliest evidence of FXR-microbiota crosstalk was discovered in a study involving the treatment of the mice with antioxidant tempol, leading to the decrease of the *genus Lactobacillus* and bile salt hydrolase (BSH) and eventually the accumulation of intestinal Tauro-β-muricholic acid (T-β-MCA), an FXR antagonist (Li et al. 2013). Another report showed that the gut microbiota regulates bile acid metabolism and inhibits the synthesis of BAs in the liver by regulating the expression of fibroblast growth factor 15 (FGF15) in the ileum and cholesterol 7α-hydroxylase (CYP7A1) in the liver. Tauro-conjugated beta and alpha-muricholic acids were also identified as the antagonists of FXR (Degirolamo et al. 2014; Gonzalez et al. 2016; Sayin et al. 2013). These discoveries provided the evidence of the potential connection between the microbiota and FXR.

Microbiota-FXR-FGF is a typical pathway of the gut microbiota-FXR-host axis. FXR targets FGF15 in mice and FGF19 in humans, respectively (Al-Khaifi et al. 2018). This pathway is related to obesity. The treatment of obesity includes the use of weight loss pills (Pathak et al. 2018) and Bariatric Surgery (Albaugh et al. 2017; Bozadjieva et al. 2018), etc. As shown in the reports, FXR-microbiota showed obesity promoting activity by increasing fatty acid transportation (Parséus et al. 2017), which was contrary to the previous cognition of FXR (Fang et al. 2015). In the intestine, the gut microbiota modulates the activity of FXR by regulating bile acid metabolism. The level of T-β-MCA, an antagonist of FXR, is regulated by BSH, an enzyme expressed in *Lactobacillus, Bacteroides, Clostridium* and *Bifidobacterium* in the gut microbiome. In some cases, for example, tempol treatment in mice, decreased these bacteria and BSH activity in the intestine and increased T-β-MCA level, and then inhibited FXR activation, resulting in suppressing the synthesis of ceramide to prevent hepatic steatosis (Jiang et al. 2015), glucose intolerance and obesity (Gonzalez et al. 2016; Jiang et al. 2015; Turpin et al. 2014).

Besides, Type 2 Diabetes (T2D) associated with glucose metabolism is related to the microbiota-FXR axis (Pathak et al. 2018). One of the mechanisms by which the gut microbiota-FXR axis regulates glucose metabolism is the FXR-glucagon-like peptide-1 (GLP-1) pathway. The activation of intestinal FXR induced lithocholic acid (LCA)-producing bacteria *Acetatifactor* and *Bacteroides*, leading to producing LCA to activate Takeda G protein-coupled receptor-5 (TGR5)/GLP-1 signaling. Then the activation of TGR5/GLP-1 signaling regulated glucose metabolism, improved insulin sensitivity and promoted adipose tissue browning (Albaugh et al. 2019).

**Fig. 1:**
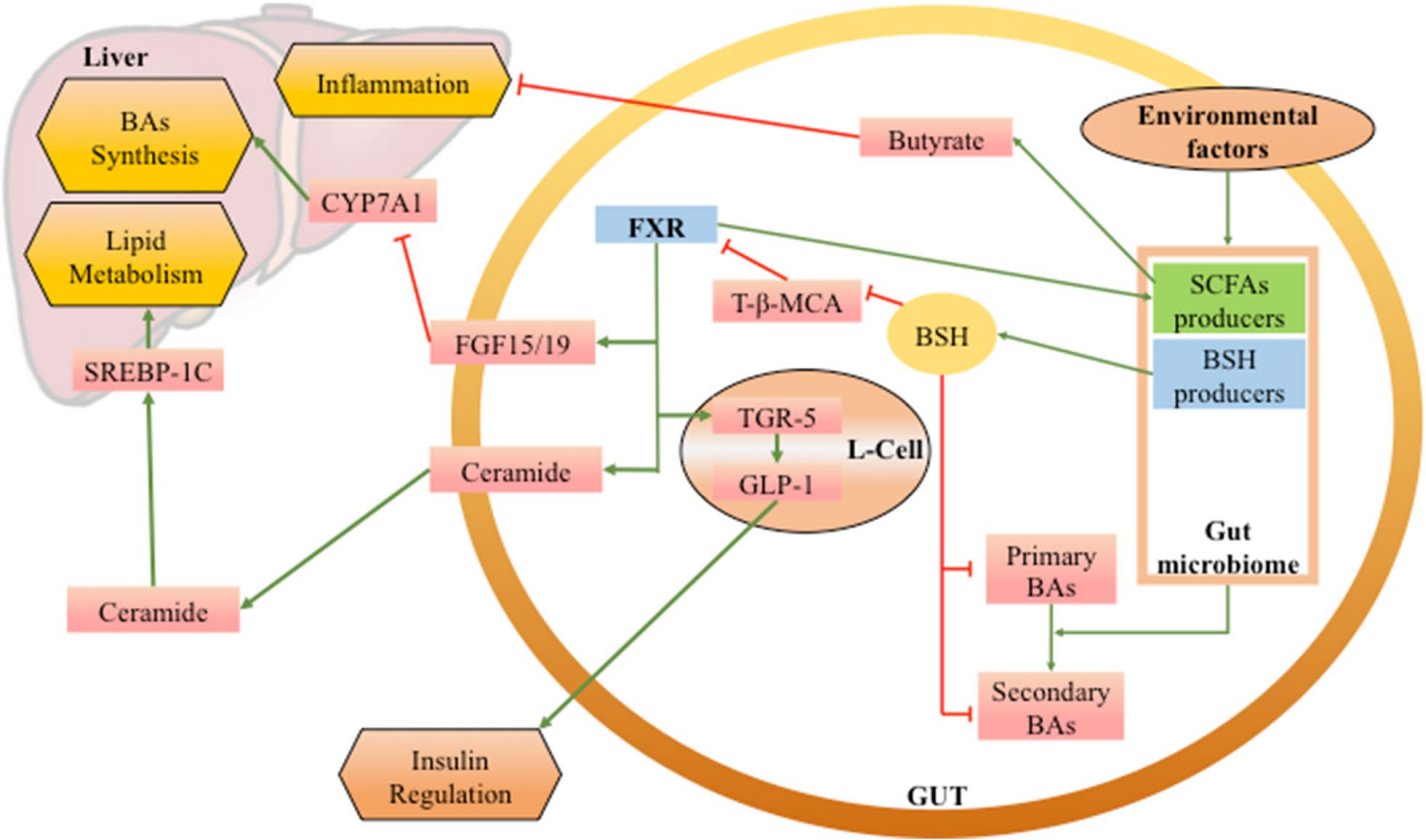
The roles of FXR in the gut microbiome-host system. Some of the mechanisms of the microbiome regulating inflammation, glucose metabolism, lipid metabolism and BA metabolism have been shown

The FXR-microbiota axis also plays a key role in the immunopathology of the gut-liver axis and IBD (Chiang and Ferrell 2018; Joyce and Gahan 2016). NAFLD is a series of liver diseases involving chronic inflammations of the liver (Chen et al. 2019). SCFAs, butyrate as one of the examples, are the products of the metabolism of the gut microbiota. Down-regulating FXR leads to the downregulation of the butyrate-generating microbes and then the decrease of the levels of butyrate, a regulator of liver inflammation (Sheng et al. 2017). Furthermore, CDCA, a typical agonist of FXR, could be changed into secondary BAs like Deoxycholic acid (DCA) and LCA by the gut microbiota. It then becomes an FXR antagonist and influences NAFLD (Jiao et al. 2018). In IBD, the FXR-FGF axis is also effective through the function of the gut microbiome. The gut microbiome modulates BA pool through the producers such as *Firmicutes*, *Bacteroidetes* and *Actinobacteria* to regulate FXR activation (Baars et al. 2015). Then activation of the FXR-FGF19 axis in a murine model of intestinal inflammation could bona fide provide positive changes in BA metabolism with consequent reduction of intestinal inflammation and modulation of microbiota (Duboc et al. 2013; Gadaleta et al. 2020). These roles form the gut microbiome-FXR-FGF cyclic regulation mechanism in IBD. On the other hand, the excess activation of FXR and type I interferon (IFN)-I signal within intestinal epithelial cells after a Western diet consumption can induce Paneth cell defects and destroy intestinal homeostasis, affecting the gut microbiota in the host (Liu et al. 2021). Thus, FXR and the gut microbiome have a relationship of mutual influence and regulation in the intestine. The functions of FXR in the microbiota-host system have been reported more than the other NRs. We have summarized a part of the findings in Fig. 1 as a signaling map.

## Gut microbiota and PPARs

PPARs, including PPARα, β, γ, δ, are a series of nuclear receptors sub-family and were first identified and cloned in 1990 (Issemann and Green 1990). Early research found that PPARs could be activated by peroxisome proliferators and fatty acids, and then regulate the metabolism of the fatty acids and carcinogenesis (Auwerx 1992; Green 1992). With the increasing number of reports, PPARs have been known as essential regulators that play key roles in various physiology and pathology processes related to not only lipid and fatty acids metabolism, and tumor generation, but also glucose metabolism, inflammation, and immunology (Mirza et al. 2019).

PPARs, as the typical model NRs, play the important roles in the host-gut microbiome crosstalk, and they have been identified as the enteric epithelial homeostasis mediators (Gao et al. 2018a). Angiopoietin-like 4 (ANGTPL4) and adipose differentiation-related protein (ADRP) are both the target genes of PPARγ, which in turn could be up-regulated by SCFAs (Butyrate and propionate) and the products of *Prevotella* and *Atopobium*. The mechanism underlying this process is the phosphorylation of PPARγ through extracellular signal-regulated kinase (ERK) signaling pathway (Nepelska et al. 2017). And the PPAR-γ signal activated by butyrate can inhibit the expression of nitric oxide synthase 2, reduce the synthesis of inducible nitric oxide synthase to limit luminal nitrate availability, and then inhibit dysbiotic *Enterobacteriaceae* expansion (Byndloss et al. 2017).

Similar to the FXR, PPARs are also involved in lipid and glucose metabolism, and thus, associated with obesity and diabetes (Gao et al. 2018b; Mishra et al. 2016). According to a recent report, *Bacteroides*, a member of the microbiome in the gut, seems to play key roles through the host-microbiome crosstalk in regulating diseases related to glucose and lipid metabolism (Zhang et al. 2016). In this process, PPARγ and PPARα were found at the abnormal expression levels (Nihei et al. 2018), which increased the sensitivity of insulin and prevented obesity (Yang et al. 2017).

PPARs are also involved in microbiome-related immune metabolism and inflammation of the gut-liver axis, such as IBD, Alcoholic Fatty Liver Diseases (AFLD) and NAFLD (Mirza et al. 2019; Sharma et al. 2015). PPARα has been confirmed to regulate the expression of Interleukin-22 (IL-22), Regenerating islet-derived III β (RegIIIβ), Regenerating islet-derived III γ (RegIIIγ or REG3G) and calprotectin in the innate immune cells, thus, mediating the gut mucosal immunity (Manoharan et al. 2016). Moreover, PPARs are involved in the process of the differentiation of anti-inflammatory M2 macrophages. The underlying mechanisms include the production of α-Linolenic acid (ALA) by gut lactic acid bacteria and subsequent induction of the macrophages through G-protein-coupled receptor 40 (GPCR40) signaling (Ohue-Kitano et al. 2018). PPARs also play key roles in preventing the exogenous infection caused by *Escherichia* and *Salmonella* (Byndloss et al. 2017; Gillis et al. 2018). As far as the acute inflammatory response is concerned, some of the probiotics produced by the gut microbiome such as *Lactobacillus casei Zhang* could reduce the inflammation (Wang et al. 2016b). Moreover, the gut microbiome could mediate PPARγ-driven liver circadian clock reprogramming (Murakami et al. 2016). The hepatic physiology follows a daily rhythm and the perturbation of the liver clock results in metabolic disorders such as NAFLD (Crespo et al. 2021) and even liver cancer (Mazzoccoli et al. 2019) through regulating rhythm gene expression and the rhythm-related signaling pathways. Thus, the gut microbiome-PPARγ axis may mediate the circadian clock to affect liver diseases such as NAFLD and cancer.

## Gut microbiome and AHR

AHR was discovered in the 1970s, and identified as a xenobiotic sensor mediating the toxicity of 2,3,7,8-tetrachlorodibenzo-p-dioxin (TCCD) initially (Guenthner and Nebert 1977; Lee et al. 2017). After more than 30 years of research, more and more functions of AHR have been identified, including the detoxing mediator, aromatic molecule (such as tryptophan, purine), metabolic regulator, cancer regulator, immune-regulator, barrier organ or cell regulator, etc. (Esser 2016; Esser and Rannug 2015; Murray et al. 2014). Similar to FXR and PPARs, AHR is associated with the gut microbiome due to the crosstalk of the host and microbiome. The ligands of AHR include SCFAs (especially butyrate) which are known as the products of the gut microbiome (Marinelli et al. 2019). Besides, indole, another typical agonist of AHR, is also a product of the host-microbiome metabolism (Rothhammer et al. 2016).

AHR is a regulator of inflammation and immune metabolism especially in the central nerve system (CNS), intestinal barrier, lymphatic system, and alcoholic hepatitis. Ethanol decomposition leads to the abnormal states of indole-3-acetic acid (IAA)-IL-22-REG3G signaling pathway and results in the transfer of the bacteria to the liver, thus, leading to the inflammation of the liver (Hendrikx et al. 2018). Apart from liver diseases, IBD and CNS immune regulation is also related to AHR (Hendrikx et al. 2018; Lee et al. 2017). The main underlying mechanisms are Caspase recruitment domain-containing protein 9 (CARD9) and IL-22 signal or cluster of differentiation 4- Lymphocyte-activation gene 3 (CD4^+^-LAG-3) pathway (Kadowaki et al. 2016; Lamas et al. 2017). Besides, IBD is associated with the lymphatic system, which is mainly related to group-3-innate lymphoid cells (group-3-ILC)-induced cellular immunity (Guo et al. 2015; Qiu and Zhou 2013; Wagage et al. 2015).

Tryptophan (Trp) metabolism of the gut microbiome also plays a key role in IBD (Lamas et al. 2016b; Longhi et al. 2017). This is related to the regulation of the gut barrier integrity and the mucosal reactivity by AHR (Singh et al. 2019; Zelante et al. 2013). Trp in the intestine could be changed to AHR ligand (including indole) by the microbiome metabolism and then can regulate multiple pathways, including IL22 signaling, AHR-xenobiotics metabolism, GLP-1 secretions, and gut-brain (CNS) axis (Agus et al. 2018). Besides, purinergic metabolism is another process that is regulated by AHR. This is associated with the immune metabolism of the intestine through targeting Cluster of Differentiation 39 (CD39) in IBD (Longhi et al. 2017).

## Gut microbiome and VDR

VDR was identified as a transcription factor belonging to the nuclear receptor superfamily (Makishima 2017); this was confirmed in diverse sources such as chicken intestine (Wecksler and Norman 1980), mouse kidney (Colston and Feldman 1980), and human breast cancer cell lines (Findlay et al. 1980). Vitamin D (1,25(OH)2D3), the ligand of VDR, a sterol and prohormone, is obtained from inactive vitamin D [25(OH)D3] (Del Pinto et al. 2017). Vitamin D regulates various physiological and pathological processes such as phosphate and calcium cycle, inflammation, immune response, and cancer, etc. (Colotta et al. 2017; Shang and Sun 2017). After activation by vitamin D, VDR could bind with RXR and forms a heterodimer just like FXR (Yoon and Sun 2011). The relationship between VDR and the gut microbiome could be understood by some phenotype research. However, the underlying mechanism is mostly unknown due to the lack of relevant studies. So far, it has been identified that the VDR-Vitamin D axis plays the key roles in IBD (Del Pinto et al. 2017), gut Vitamin D regulation (Barbáchano et al. 2017), microbiome homeostasis, epithelium and mucosal regulation (including immune regulation) (Kanhere et al. 2018), sterol metabolism (Ridlon and Bajaj 2015), and autophagy regulation (Sun 2016).

Cooperating with the gut microbiome, the Vitamin D-VDR axis plays the key roles in intestine inflammation, certainly in IBD, through multiple signaling pathways including NF-κB, Mitogen-activated protein kinase (MAPK), Toll-like receptor (TLR), epidermal growth factor receptor (EGFR), etc. (Wu et al. 2010; Yoon and Sun 2011). And the function of VDR in intestine inflammation regulation is associated with epithelium and mucosa through these mechanisms, which also acts as key roles in cystic fibrosis (Kanhere et al. 2018). In a report of VDR^−/−^ colon inflammation mouse model, VDR knockout mice showed upregulation of IFN-γ^+^ and Interleukin 17^+^ (IL17^+^) T cells (Th1 and Th17) that results in the mucosa inflammation and the apoptosis of epithelium cells (He et al. 2018). Besides, *Lactobacillus casei Zhang* could inhibit colon cancer through multi-signaling (including adenosine monophosphate-activated protein kinase (AMPK) signaling pathway) along with Vitamin K2 (Zhang et al. 2017). At the same time, downregulation of VDR leads to the abnormal autophagic activity and the abnormal states of the gut microbiome by reducing the level of autophagy related 16 like 1 (ATG16L1), which is associated with intestine inflammation (Jin et al. 2015; Sun 2016).

## Prospect

The gut microbiome has been identified as a subsystem that plays the key roles in various complex physiological and pathological processes. NRs have also been confirmed as intermediators in the microbiome-host axis; however, the signal and pathway map is incomplete. As oral medication is one of the most efficient methods in clinical treatment, the gut microbiome could be a medium medicine targeting the NRs. As the roles played by NRs are known to be complex, straightforward targeting of NRs might lead to serious toxic side effects. For example, the previous report has shown that obeticholic acid, a ligand of FXR, leads to an unfavorable serum lipid profile with the increase of total cholesterol and low-density lipoprotein cholesterol and the decrease of high-density lipoprotein cholesterol (Massafra et al. 2018; Mudaliar et al. 2013). Targeting PPARγ can relieve insulin resistance and promote adipogenesis, which makes the role of PPARγ self-contradictory in the treatment of T2D (Lehrke and Lazar 2005). And thiazolidinediones (TZDs, the ligands of PPARγ) also show a huge risk of clinical application (Ahmadian et al. 2013). However, due to the gut microbiota-NRs-host axis and the regulation of the gut microbiome by diet therapy, indirectly targeting NRs through diet change would be an ideal way to reduce the side effects of NRs caused by the direct ligand application. Hence, the activation of the gut microbiota-NRs-host axis may be used for avoiding some of the risks associated with the toxic effects induced by the NR ligand treatment.

Besides, targeting a single factor seems ineffective in some of the diseases with complex pathological processes. As a complex system, the gut microbiome impacts the host physiology processes through targeting multi-signaling pathways and multi-NRs in the due course. It implies that many pathological processes are due to a combination of factors rather than a single path. This could be a topic of potential research in the future.

## Conclusion

In summary, NRs are the important mediators between the gut microbiota and the host. The functions of NRs, as the important regulators, in the host can be influenced by the gut microbiome. On the other hand, the condition of the gut microbiome is also affected by NRs, just as FXR has effects on the gut microbiome in IBD (Duboc et al. 2013; Liu et al. 2021). NRs can be identified as a bridge between the gut microbiome and the host.

## Data Availability

No supporting data.

## Abbreviations

ADRP: Adipose differentiation-related protein
AFLD: Alcoholic fatty liver disease
AHR: Aryl hydrocarbon receptor
ALA: α-Linolenic acid
ALD: Alcoholic liver disease
AMPK: Adenosine monophosphate-activated protein kinase
ANGPTL4: Angiopoietin-like 4
ATG16L1: Autophagy related 16 like 1
BAs: Bile acids
BSH: Bile salt hydrolase
CAR: Constitutive androstane receptor
CARD9: Caspase recruitment domain-containing protein 9
CD4: Cluster of differentiation 4
CDCA: Chenodeoxycholic acid
CNS: Central nerve system
CYP7A1: Cholesterol 7α-hydroxylase
DCA: Deoxycholic acid
ERK: Extracellular signal-regulated kinase
FGFs: Fibroblast growth factors
FXR: Farnesoid X receptor
GLP-1: Glucagon-like peptide-1
GPCR40: G-protein-coupled receptor 40
IAA: Indole-3-acetic acid
IBD: Inflammatory bowel disease
IFN-γ: Interferon gamma
ILC: Innate lymphoid cells
ILs: Interleukins
JNK: C-Jun N-terminal kinases
LAG-3: Lymphocyte-activation gene 3
LCA: Lithocholic acid
LXR: Liver X receptor
MAPK: Mitogen-activated protein kinase
MC-LR: Microcystin-LR
NAFLD: Non-alcoholic fatty liver disease
NALD: Non-alcoholic liver disease
NF-κB: Nuclear factor kappa-light-chain-enhancer of activated B cells
NRs: Nuclear receptors
PPARs: Peroxisome proliferator-activated receptors
PXR: Pregnane and xenobiotic receptor
RegIII: Regenerating islet-derived III
RXR: Retinoid X receptor
SCFAs: Short chain fatty acids
SREBP-1: Sterol regulatory element-binding protein 1
T2D: Type 2 diabetes
TCCD: 2,3,7,8-Tetrachlorodibenzo-p-dioxin
TGR5: Takeda G protein-coupled receptor-5
VD: Vitamin D
VDR: Vitamin D receptor
